# Production of Monoclonal Antibodies in Plants for Cancer Immunotherapy

**DOI:** 10.1155/2015/306164

**Published:** 2015-10-15

**Authors:** Ghislain Moussavou, Kisung Ko, Jeong-Hwan Lee, Young-Kug Choo

**Affiliations:** ^1^Major of Nano-Bioengineering, College of Life Sciences and Bioengineering, Incheon National University, Incheon 406-840, Republic of Korea; ^2^Department of Medicine, College of Medicine, Chung-Ang University, Seoul 156-756, Republic of Korea; ^3^Department of Biological Science, College of Natural Sciences, Wonkwang University, Iksan, Jeonbuk 570-749, Republic of Korea; ^4^Institute for Glycoscience, Wonkwang University, Iksan, Jeonbuk 570-749, Republic of Korea

## Abstract

Plants are considered as an alternative platform for recombinant monoclonal antibody (mAb) production due to the improvement and diversification of transgenic techniques. The diversity of plant species offers a multitude of possibilities for the valorization of genetic resources. Moreover, plants can be propagated indefinitely, providing cheap biomass production on a large scale in controlled conditions. Thus, recent studies have shown the successful development of plant systems for the production of mAbs for cancer immunotherapy. However, their several limitations have to be resolved for efficient antibody production in plants.

## 1. Introduction

Cancer is a class of diseases involving uncontrolled abnormal cell growth and spreading [1]. These cancer cells originate from the same clone, initiating malignant tumor cell growth capable of out-of-control proliferation. During cancer development, some cells may migrate from their place of origin, that is, metastasize, and cause secondary tumors in other parts of the body. Due to these characteristics, cancer should be detected as early as possible. There are more than one hundred known different types of cancer, and each can be classified by the type of cell that was initially affected. Cancer treatments, including chemotherapy, major surgery, and other long-term treatments, make cancer the most expensive disease to treat, and the cost continues to increase; in addition to the economic burden, the social burden associated with cancer is also huge. Among all treatments, the use of chemotherapeutic agents only provides minimal survival benefits due to several factors such as drug resistance, side effects, and toxicity. The incidence of cancer is increasing in both developing and developed countries; thus the development of new and cheap molecules for cancer chemotherapy is necessary [2]. As such, the development of natural or synthetic agents, including immunotherapeutic proteins, to prevent or suppress cancer progression has recently been recognized as a field with enormous potential [3].

Recently, experimental and clinical studies have revealed the mechanisms of antibody-mediated killing responses against tumor cells that induce effective, consistent, and durable cancer suppressing activities. Indeed, the presence of spontaneous or induced tumor cells in the body triggers antitumor responses. One of these antitumor responses is the generation of a large number of antibodies for direct tumor-cell killing, immune-mediated tumor-cell killing, and vascular and stromal ablation [4]. Therefore, people have tried to understand how to design monoclonal antibodies (mAbs) that specifically recognize a certain antigen, found on the surface of cancer cells, to enhance the mAb activities promoting such antitumor mechanisms. Each mAb recognizes one particular tumor-associated antigen, working in different ways depending on the antigenic targets on the different types of cancer cells. There are three main types of mAbs, which work in different ways: trigger the immune system to attack cancer cells, block the signals telling cancer cells to divide, or carry drugs or radiation to cancer cells [5].

Despite the highly efficient therapeutic activities of mAbs for cancers, mAb therapy has not been widely applied due to high production costs, potential human pathogen contamination, and limited scalability of the mammalian cell-mediated system. Therefore, heterologous production platforms with cost-effectiveness, safety, and scalability have been developed using other bioorganisms such as bacteria, insects, yeast, and plants [6–13]. Among them, the use of plants for the production of such anticancer mAbs is attractive due to the low production cost, scalability, and ability to assemble and modify multimeric mAb proteins [13–18]. Therefore, plant production systems are considered to have the potential to compete with other systems, such as bacteria, yeast, or insect and mammalian cell cultures, for the production of mAbs [5].

## 2. mAb Structure and Anticancer Mechanism

There are five classes of antibodies (immunoglobulins) defined by the structure of the constant region of the heavy chain: IgG, IgA, IgM, IgD, and IgE. These five classes of antibodies are further differentiated according to their composition, charge, and molecular weight. Among these classes, IgG and IgM are the ones mainly involved in various therapeutic applications. Furthermore, antibodies are composed of two identical light and heavy polypeptide chains linked together. For the IgG molecule, the variable amino acid terminal sequence domains of light and heavy chains are termed V_L_ and V_H_, respectively, whereas the corresponding constant sequence domain of each chain is termed C_L_ and C_H_ [18]. Thus, the light chain has two intrachain disulfide bonds, one in the V_L_ and the other one in the C_L_, whereas the heavy chain, which is twice as long, has four intrachain disulfide bonds. The variable and constant regions of antibody have two important functions: one is binding to the specific antigen to prevent pathogens from entering or damaging cells and the other is recruiting various immune-related cells and molecules to disrupt the functions of antigens and destroy tumor cells or pathogens [10].

Many studies have revealed that mAbs can trigger cytotoxic reactions through the complement system and/or the activation of effector cells, including natural killer cells and macrophages, to destroy tumor cells. The antitumor mechanisms are mainly antibody-dependent cellular cytotoxicity (ADCC) and complement-dependent cytotoxicity (CDC). Through ADCC, the immune cells are educated to kill tumor cells [5, 35]. According to Vitetta and Uhr [36] and Vuist et al. [37], through CDC, mAbs bound to tumor cells trigger transmembrane signals that inhibit tumor growth, consequently leading to apoptosis [36, 37]. Antibodies, even not labeled with any drug or radioactive material, show significant efficacy in some cancer treatment, including breast and colorectal cancers [5].

Antibodies can inhibit the activity of foreign molecules, pathogens, or tumor cells due to the affinity of their variable binding regions for targeted antigens. They also have effector functions such as ADCC, complement action, and phagocytosis due to the efficient interactions between their Fc region and Fc receptors of immune cells, including the binding property of mAb to the targeted antigens [5, 35]. To enhance the mAb antigen specificity and binding affinity, the amino acid sequences can be modified at the mAb antigen binding site [38, 39]. In addition, mAb affinity can be improved by modifying the glycan structure and the degree of glycosylation [40].

Plants have been used for mAb production, with the tobacco plant being the first and the main one [14]. The mAbs expressed from tobacco plants can fully recognize cancers cells [16, 29]. An anticolorectal cancer mAb, mAb CO17-1A (IgG_2a_), binds the tumor-associated antigen GA733, which is highly expressed on the surface of human colorectal carcinoma cells [41]. mAbs are efficient in treating metastases and in preventing the recurrence of colorectal cancer in high-risk patients [42, 43]. The full-size recombinant mAb CO17-1A has been expressed in a plant system through a tobacco mosaic virus vector [44]. The plant-derived mAb CO17-1A heavy and light chains were assembled to bind the recombinant antigen GA733 and also specifically bind to human SW948 colorectal carcinoma cells expressing the antigen GA733 [16, 18]. The plant-derived mAb (mAb^P^) was as effective as mAb^M^ CO17-1A in inhibiting the tumor growth of human colorectal carcinoma SW948 cells xenotransplanted into nude mice. Furthermore, antibreast cancer mAb (mAb BR55-2) recognizes the Lewis Y oligosaccharide antigen (LeY), which exists predominantly on breast, lung, ovary, and colon cancer cells [45–47]. Steplewski et al. [48] reported that murine mAb BR55-2 (IgG_2a_) inhibits tumor growth and kills human cancer cells xenografted into nude mice [49]. Brodzik et al. [29] successfully expressed and assembled a functional mAb BR55-2 (IgG_2a_) specific to LeY oligosaccharide in transgenic tobacco plants with low alkaloid content (*Nicotiana tabacum* cv. LAMD609) [29]. Similar to mAb^M^, the mAb^P^ bound specifically to both breast and colorectal cancer cells [29]. A single plant can express two different mAbs to recognize two different antigenic targets [50]. Both antirabies virus human mAb (mAb^H^) and anticolorectal cancer mAb^M^ CO17-1A (mAb^M^ C) were successfully expressed in a single transgenic plant.

## 3. Plant Systems for mAb Production

mAbs have been often produced in different expression systems such as yeast, insect cells, and mammal cells. Recently, different mAbs and their derivatives have also been expressed in plants [51]. In most plant systems used for large-scale mAb production, the transformed plants, which act as bioreactors, are cultivated* in vitro*, allowing the regeneration of mature plants and the propagation of plant cells as a cell-suspension culture platform. These plant systems help manufacture plant biomass* in vitro*, including leaves, stems, and roots, and the mature plants can be transplanted and grown* in vivo* (in soil pots) [52]. Thus, the plants are different from other cell-culture production systems described above in terms of the flexibility for use in both* in vitro* and* in vivo* platform conditions. Tables 1 and 2 show the comparison between heterologous bioexpression systems and between transgenic mammalian cells and transgenic plant systems, respectively.

Plant systems such as tobacco, alfalfa, and some other species have been developed as they are the most accessible and common sources of leaf biomass [53]. Maize and soybean can produce and accumulate mAbs in the seeds. Some vegetable plants have relatively high total soluble protein levels, which might be beneficial for recombinant protein expression [54]. Among vegetable plants, the leaf biomass of Chinese cabbage has the highest total soluble protein level compared to others, making it a candidate bioreactor to produce recombinant therapeutic proteins. Tobacco has the major advantages such as high leaf biomass yield and rapid scale-up through easy seed production, when compared to other plant species [16]. In a recent report, the expression level of recombinant proteins in tobacco stems was similar to that of leaves, thus suggesting that the whole tobacco plant biomass can be used for production of recombinant therapeutic proteins, eventually increasing the upstream production cost efficiency [52]. Additionally, tobacco is a nonfood, nonfeed plant that has been well characterized as an expression system excluding human pathogen contamination, which reduces biosafety concerns. However, tobacco contains nicotine or other toxic alkaloids, which need to be removed using an additional extraction step [55]. Furthermore, tobacco produces heterogeneously N-glycosylated antibodies due to the different place distribution of antibody in the secretory pathway, which may cause difficulties in controlling the quality of the antibody produced [40, 43, 56, 57]. Alfalfa has some benefits such as a high yield of biomass and a homogeneous glycan structure [57]. However, alfalfa is used as animal feed, for a source of oxalic acid. Arabidopsis, however, can be considered as a nonfeed plant expression system with high total soluble protein level in leaf and stem [54]. Maize is superior in terms of biomass yield, but its* in vitro* transformation and manipulation are recalcitrant. In plant expression systems, leaves and seeds have both advantages and disadvantages, and both seem appropriate for the expression of all targeted proteins. Leaves with an active and complex metabolism have high protease activities toward degrading certain proteins [58]. The seeds have lower water content, providing a stable protein accumulation. However, they need a large amount of energy to grind during the purification downstream processing. Nevertheless, many transgenic plants producing recombinant proteins have been developed for the process of being commercialized. Tables 3 and 4 show the recombinant protein expression in leaves (tobacco) and seeds (rice) and transgenic plants used in the production of antibodies, respectively [59, 60]. The selection of plant species should be carefully considered for successful production of antibodies, since each plant species has its own physical and physiological characteristics affecting the expression and glycosylation of recombinant glycoproteins [16]. Despite showing a potential for therapeutic mAb production, plants are not perfect production systems for biopharmaceutical proteins, due to the incapability of human N-glycosylation [8, 40]. In nature, the N-glycan structures of glycoproteins are diverse in the different organisms such as insects, yeast, plants, and mammals. Plants have their own N-glycosylation apparatus to generate plant specific glycans. Thus, the N-glycosylation in plant cells differs from that of mammalian cells [61]. Four types of N-glycan structure exist in plant: oligomannose, complex, hybrid, and paucimannose [18]. All these glycans harbor a common core structure, Man_3_GlcNac_2_, where the additional sugar residues attached include a *β*(1,2) xylose and an *α*(1,3) fructose residue, which are often considered as allergenic epitopes inducing IgE [62–64]. Additionally, plants do not have sialic acid residues on their glycan structures, which is essential for glycoprotein stability [19, 33]. In nature, after proteins enter into the endoplasmic reticulum (ER) through a signal peptide, the proteins are folded, assembled, and N-glycosylated, and the glycosylated proteins are then secreted outside passing through the Golgi complex in plant cells [18, 65]. Therapeutic proteins are mainly N-glycosylated, and thus glycan structures on the proteins can affect their stability, folding, and biological activity [8, 40]. Glycosylation affects vital biological characteristics, including immunogenicity, allogenicity, and interactions between ligand and receptor proteins [16, 58]. Thus, a certain N-glycan structure on antibodies produced from any heterologous expression system is required to keep their intending therapeutic effects similar to the parental antibody [16]. The afucosylated glycosylation structure on Fc regions of mAb enhances the interaction between Fc regions and Fc receptors, consequently increasing ADCC [66]. The antibody-mediated tumor inhibition is mainly due to ADCC [67]. Transgenic tobacco plants have been successfully obtained to express both anticolorectal cancer mAb CO17-1A to secrete to the outer membrane of plant cells and the mAb, including a KDEL sequence, a ER retention signal to target the accumulation of mAb inside ER in plant cells [18]. Both mAb^P^ CO17-1A and mAb^P^ CO17-1A with KDEL were compared with mAb^M^ CO17-1A in N-glycan structures and* in vitro* biological activities. In tobacco plant, the mAb CO17-1A with KDEL was accumulated higher compared to mAb CO17-1A without KDEL, suggesting that the ER localization enhances the level of mAb CO17-1A in plants. It was also reported that ER localization could alter the glycan structure of antibodies to an oligomannose-type of glycan structure, consequently influencing its function, such as the interaction between Fc regions and Fc receptor for antitumor activity [18]. For humanization of N-glycan structures of recombinant human erythropoietin (hEPO) proteins in plant, mammalian *β*(1,4)-galactosyltransferase (GalT) and *α*(1,6)-fucosyltransferase genes were successfully expressed to generate hEPO with humanized N-glycans at great uniformity in a mutant plant without *β*(1,2)-xylosyltransferase and *α*(1,3)-fucosyltransferase gene expression [21]. Glycoengineering in plants has been currently studied as a powerful tool to produce recombinant anticancer mAbs with tailor-made N-glycan structures.

## 4. Expression of Recombinant mAb Proteins in Plants

**Figure 1:**
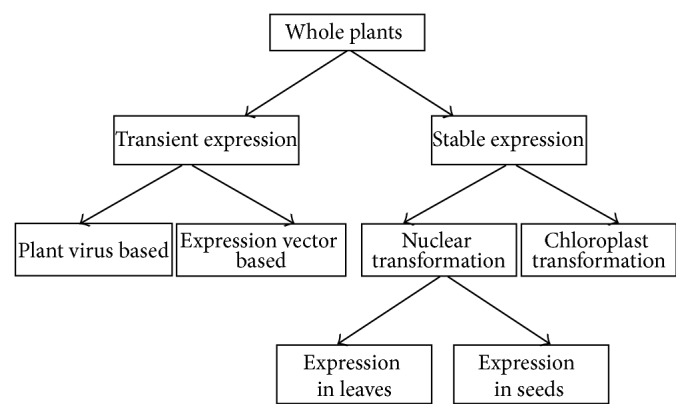
Choice of transgene expression system.

Plants can be regenerated from somatic cells due to their pluripotency [68]. Plant cells appear as a fundamental unit in the process of transgenic lineage plants creation. Additionally, protocols to transfer the recombinant antibody genes into plant cells with the hard pectocellulose wall acting as barrier are essential for the recombinant antibody expression in plants. There are two different transformation protocols with stable and transient expression [30, 69]. The first one is an expression protocol for the stable genetic transformation, where agrobacterium and particle bombardment, which are biological or physical methods, respectively, have been currently applied to allow the penetration of cDNA encoding both heavy and light chains of antibodies directly into the plant cells and to stably insert the cDNA into the genomes of the plants [30]. The heavy and light chain genes can be introduced separately into individual plants [31]. Plants highly expressing each heavy or light chain can be selected and crossed to generate transgenic lines expressing both heavy and light chains. This crossing approach can be used to express multiple antibodies and antigens with glycomodification [50]. The gene can be inserted into the chloroplast genome to generate chloroplast transgenic plants expressing and properly folding antibodies with disulfide bonds [70]. Many advantages can be obtained from chloroplast transformation including the lack of transgene pollen transmission due to the lack of plastids in mature pollen and high expression levels with highly polyploidy genomes. Indeed, a large number of plastids carrying multiple transgene copies can exist in a cell, resulting in very strong chloroplast expression of up to 25% soluble proteins. Additionally, position effects or gene silencing does not exist in chloroplasts. Thus, if the proper glycosylation is potentially built in the chloroplast, the chloroplast transformation might emerge as a potential stable expression system for anticancer antibodies [70]. Agroinfiltration and recombinant plant viruses have been applied as transient expression systems for mAb production. Agroinfiltration systems can successfully produce mAbs on a large scale [34, 71]. Agroinfiltration system has been successfully applied to generate multiantennary N-glycans that mainly exist in mammalian-derived glycoproteins [72, 73]. Plant viral vectors can be used for the transient expression of mAb more rapidly than transgenic plants. Thus, the viral vectors can quickly be inoculated to rapidly produce single-chain antibody (scFv) customized for cancer patients with unique epitopes [74]. Additionally, full-size mAbs have been expressed in* Nicotiana benthamiana* through two Tobacco Mosaic Virus (TMV) vectors carrying heavy and light chains [44, 74]. However, the plant viral system requires virus inoculation to leaf or stem every time due to its transient gene expression in plant and, thus, a frequent gene mutation occurs during virus replication unlike transgenic stable expression system [75]. Thus, the choice of gene expression technique and production system should be properly pondered (Figure 1) [76].

## 5. Purification of mAbs from Plants

Purification of antibodies expressed in plants has been successfully established using protein A- or G-based affinity chromatography [77]. For purification, the plant tissues must be homogenized to disrupt the cell walls releasing the cell debris, noxious chemicals, and contaminants, which should be removed using purification processes [30]. The purification processes are challenging due to their large-scale factor and, thus, the affinity-matrix column purification systems cannot avoid clogging problems in the column caused by the plant cell wall debris being left over during biomass homogenization and removal processes [77]. In addition, the protein A column application is limited by its high cost. Protein A has been fused with oleosin to express protein A-oleosin oil bodies in transgenic oleaginous plant seeds, which can capture the antibody in the oil-body phase, where the antibody is mixed with protein A-oleosin [78]. The antibody captured from the oil-body can be partitioned from the impurity-carrying aqueous phase through simple centrifugations and eventually eluted from the oil bodies. This protein A-oleosin fusion technology based on simple mixing and phase separation can be applied as an inexpensive and scalable process for antibody purification in the plant expression system [79, 80]. Several other fusion protein strategies have been developed to improve production level of recombinant proteins together with efficient purification in plant. Zera, a domain of prolamine-rich (gamma) maize storage protein accumulated inside the ER, can form stable supramolecular aggregates of polyproline structure bodies in plant cells, which allow the high accumulation of recombinant proteins in the ER and, thus, facilitates protein recovery through simple homogenization and centrifugation, enabling efficient purification [81]. Elastin-like polypeptides, repetitive biopolymers exist as soluble forms below their transition temperature and aggregate into micron-scale coacervates above the transition temperature [82]. The recombinant proteins fused into elastin-like polypeptide tags can be purified through the selective removal of both soluble and insoluble contaminants, without chromatography [83]. Hydrophobin, which is a small and surface-active fungal protein, is another applicable fusion protein enhancing the accumulation of its fusion recombinant protein through protein body formation in plants and altering the hydrophobicity for efficient purification, using a surfactant-based aqueous two-phase system (ATPS) [84–86]. These protein fusion technologies are promising tools to allow for high accumulation and low-cost purification of recombinant antibody in plant expression systems.

## Figures and Tables

**Table 1:** Comparison of heterologous bioexpression systems.

Expression systems	Yeast	Insects	Mammalian cells	Plants
Production cost	Medium	Medium to high	High	Low
Maintaining cost	Cheap	Expensive	Expensive	Cheap
Protein yield	High	Medium to high	Medium to high	High
Gene size restriction	Unknown	Unknown	Limited	Not limited
Therapeutic risk	Unknown	Unknown	Yes	Unknown
Glycosylation	High mannose	Mannose terminal	Correct	Plant specific
Safety	Unknown	Medium	Medium	High
Time required	Medium	Medium	High	Medium

**Table 2:** Comparison between mammalian cell and plant systems.

Characteristics	Mammalian cells	Plants
Advantages	(i) Posttranslational modifications of proteins similar to the human (ii) Relatively high production capacity (easy collection and attractive yield)	(i) High production capacity (ii) Cost/attractive yield (iii) Increased viral safety (iv) Easy production (v) High uniformity of production through generation

Inconveniences	(i) Viral safety (species barrier) (ii) Risk of contamination (biological and physic chemical) (iii) Difficulty to grow	(i) Glycosylation and posttranslational modification (ii) Culture parameter being uncontrollable (iii) Risk of contamination (soil bacterium and pollen dissemination)

**Table 3:** Comparison of recombinant protein expression in leaves (tobacco) and seeds (rice).

Characteristics	Leaves (tobacco)	Seeds (rice)
Technical feasibility	(i) Easy transformation ability (ii) Protein production in leaf stem tissues (medium level of expression) (iii) Glycosylation occurs with nuclear transformation; glycosylation occurs with chloroplast transformation, reducing flexibility of protein production	(i) Relatively easy transformation ability (ii) Stable protein storage in grain (high level of expression) (iii) Glycosylation makes high flexibility in protein production

Production feasibility	(i) Fair germplasm base available (ii) Easy purification, more difficult with tissue based production (iii) More byproduct with tissue based production	(i) Very good germplasm base available (ii) Ease of purification good if targeted to endosperm (iii) More limited byproduct with grain

Containment	(i) Seed production typically difficult; chloroplast transformation reduces dissemination by seed (ii) Minimum 1/4 mile isolation distance (iii) Seed dormancy in soil less than 2 years (iv) Crop does not persist without intervention	(i) Primarily self-fertilized (ii) Relatively lower separation requirement (iii) Presence of weedy red rice (relative) must be determined, mitigated, and monitored (iv) Seed dormancy in soil is less than 2 years (v) Crop does not persist without intervention

Environmental impact	Driven by specific protein	Driven by specific protein

Food/feed impact	(i) Nonfood or nonfeed crop; nontarget species unlikely to feed (ii) Food safety generally not established (iii) Risk driven by specific protein	(i) Primarily a food crop (ii) Rice itself is not orally toxic (iii) Risk driven by specific protein

**Table 4:** Transgenic plants used in the production of therapeutic antibodies.

Target	Transgenic plants	Antibodies	Application and specificity	Reference
Virus	Soybean	IgG against HSV-2	Treatment for HSV	[19]
Virus	Tobacco	IgG against rabies virus	Treatment for rabies virus	[10, 20]
Virus	Tobacco	IgG against Ebola virus	Treatment for Ebola virus	[21, 22]
Virus	Tobacco	IgG against HIV	Treatment for HIV	[23]
Virus	Tobacco	IgG against RSV	Treatment for RSV	[24]
Virus	Tobacco	IgG against WNV	Treatment for West Nile virus	[25]
Cancer	Tobacco	ScFv against CEA	Tumor marker and clinical test	[26]
Cancer	Rice	ScFv against CEA	Tumor marker and clinical test	[27]
Cancer	Cereals	ScFv against CEA	Tumor marker and clinical test	[28]
Cancer	Tobacco	IgG against tumor antigen Lewis Y	Treatment for breast cancer	[29]
Cancer	Tobacco	IgG against tumor antigen GA733-2	Treatment for colon cancer	[18, 30]
Bacteria	Tobacco	IgA against *S. mutans*	Prevention of dental caries	[31, 32]
Bacteria	Tobacco	IgG against *S. mutans*	Prevention of dental caries	[33]
Bacteria	Tobacco	Hybrid IgA-G	Anthrax	[34]

HSV: herpes simplex virus; HIV: human immunodeficiency virus; RSV: human respiratory syncytial virus; WNV: West Nile virus; and CEA: carcinoembryonic antigen.
